# Efficacy and safety of Shengmai injection as an adjunctive therapy on sepsis: A protocol for systematic review and meta-analysis

**DOI:** 10.1097/MD.0000000000028493

**Published:** 2022-01-14

**Authors:** Yue Han, Guofu Zhong, Xiao Chang, Mujuan Xu, Mingtai Chen, Linsheng Zeng, Ling Wang

**Affiliations:** Shenzhen Traditional Chinese Medicine Hospital, Shenzhen, Guangdong, China.

**Keywords:** protocol, sepsis, Shengmai injection, systematic review

## Abstract

**Introduction:**

Sepsis is a common life-threatening, acute and severe disease with high morbidity and mortality, which seriously endangers patient health. Shengmai injection (SMI) is typically used as an alternative treatment for sepsis patients. This investigation aimed at designing a comprehensive recollection and meta-analytical exercise for evaluating efficacy and safety-profile for employing SMI against sepsis.

**Methods:**

Multiple research literature repositories, both localized and global, were examined for randomized controlled trials of sepsis treated by SMI - from repository inception to December 2021 as a timeframe. Primary outcome measures contained 28-day all-cause mortality, while secondary outcome measures consisted of Sequential Organ Failure Assessment scorings, acute Physiology and Chronic Health Evaluation II scorings, ICU-based hospitalization length, mechanical ventilation timespan, ICU mortality rate, and adverse effects/events. RevMan V.5.3 was employed for data analyses. Two reviewers evaluated bias risks/investigation quality through Cochrane Collaboration risk of bias tool / Grades of Recommendations Assessment Development and Evaluation, separately.

**Results:**

Such a comprehensive reviewing protocol review protocol systematically and objectively analyzes the effectiveness and safety-profile of SMI for therapy against sepsis, together with providing scientific grounds for clinic-based employment for SMI.

**PROSPERO registration number:**

CRD42021245247.

## 1 Introduction

Sepsis is a common and life-threatening severe illness.^[1]^ It is a systemic inflammatory reaction syndrome caused by bacteria and other pathogenic microorganisms invading the body. In addition to systemic inflammatory response syndrome and primary infection lesions, severely infected patients often have manifestations of organ hypoperfusion.^[1,2]^ Globally, sepsis prevalence rates exponentially increased during the past few years, afflicting over 19,000,000 individuals together with resulting in almost 6,000,000 mortalities annually, posing a huge burden on social health care.^[3,4]^ Presently, the treatment of sepsis mainly relies on timely and standardized use of antibiotics and supportive treatment.^[5]^ Even though sepsis pathogenesis has been established, pharmacological treatments against sepsis have not yet achieved desirable results, and sepsis morbidity and mortality failed to be markedly reduced until now.^[6]^ Consequently, there is great demand and pressure for identifying novel therapeutic measures against sepsis, having the appropriate safety profile.

In order to provide treatment against clinical manifestations together with the deep-seated core condition etiology, traditional Chinese medicine recommends a serene and homeostatic physical condition.^[7]^ This bears benefits for curing multiple septic phases.^[8]^ Nowadays, traditional Chinese medicine is being integrated within western-nations as a corollary/second-line methodology for treating sepsis.^[8]^ The Shengmai formula (SMF), initially used within Yi Xue Yuan Li, is constituted by P. ginseng root (Renshen), Ophiopogon japonicus root (Maidong), and Schisandra chinensis fruit (Wuweizi) at a dosing ratio of 5:3:1.5. SMF is typically formulated as Shengmai powder, Shengmai san, or as Shengmai injection (SMI), to be utilized within the clinical setting.^[9]^ SMI can cure severe heart myocardial infarction, cardiogenic/toxic / hemorrhagic shock, cardiac/endocrine conditions, together with other conditions due to lack of qi/yin, having a safe adverse event profile.^[10]^ SMI is particularly indicated within clinical-setting protocols as a combinatory-treatment with antibiotics against community-acquired pneumonia.^[11]^ In vivo studies have shown that SMI acts a prophylaxis for several organs through controlling immune inflammatory, apoptotic, and energetic metabolic processes.^[12]^ SMI acts as prophylaxis for murine intestinal mucosal barriers mostly by controlling NF-κB–pro-inflammation factor–myosin light-chain kinase (MLCK)–TJ signaling. Downregulation of pro-inflammation cytokines such as interferon-γ (IFN-γ), TNF-α, and IL-2 were identified within murine serum samples, treated with SMI (1.5 mL/Kg). Occludin levels were upregulated while MLCK proteomic levels were downregulated within SMI-treated murines, in comparison to endotoxemia murine cohort.^[13]^

Presently, many clinical investigations validated effectiveness/safety profiles for SMI against sepsis.^[14,15]^ For example, clinical studies^[16]^ have shown that SMI can enhance cardiac contractility, increase cardiac output, increase blood pressure together with ameliorating tissue perfusion. Concomitantly, SMI improves patient microcirculation, ultimately enhancing patient prognosis and reduce case fatality rate.^[17]^ Therefore, we believe that SMI has the potential to treat sepsis. Notwithstanding, SMI influences over sepsis appear controversial, when viewed through scientific proof-dependent perspectives. Multiple comprehensive review articles assessed medical advantages provided by SMI therapeutics against sepsis, though such investigational outcomes proved unclear.^[18]^ Recent emerging randomized controlled trials (RCTs) focusing on SMI against sepsis were identified within scientific literature.^[14,15]^ Consequently, this investigation presents the latest systematic review aimed at evaluating effectiveness / safety profiles for SMI against sepsis.

## 2 Methods

This study is in line with Preferred Reporting Items for Systematic Review and Meta-Analysis^[19]^ procedures (supplemental appendix A).

### 2.1 Study type

 

Inclusion criteria consist of Chinese/ English written RCTs of SMI for sepsis. Exclusion criteria are medical record reports and semi-random RCTs.

### 2.2 Participants

Confirmed clinical sepsis cases, according to internationally accepted diagnostic criteria, with no form of demographic limitations.

### 2.3 Interventions

Study control cohort is provided treatment employing western-medicine protocols, while focus cohort is provided treatment using SMI, depending upon control cohort. Dosage, treatment time and manufacturer for SMI are not limited.

### 2.4 Outcome measures

#### 2.4.1 Primary outcomes

28-day all-cause mortality.

#### 2.4.2 Secondary outcomes

- The Sequential Organ Failure Assessment scores
- The Acute Physiology and Chronic Health Evaluation II score
- ICU-based hospitalization length
- Mechanical ventilation timespan
- ICU mortality rate
- Adverse effects/events.

### 2.5 Search strategy

This study analyzes PubMed, Embase, Cochrane Library, China National Knowledge Infrastructure, Web of Science, Chongqing VIP, together with Wanfang databases, covering a timeframe spanning repository inception until December 2021. Search key-words are “sepsis,” “SMI,” and “RCTs.” The key-words are converted into Mandarin/Cantonese for study identification within such repositories. Literature-search protocol regarding PubMed is depicted within Table 1.

**Table 1:** Literature-search strategy employed for PubMed repository.

Number	Search terms
#1	Sepsis [MeSH]
#2	Sepsis [Title/Abstract]
#3	#1 OR #2
#4	Shengmai injection [MeSH]
#5	Shengmai [Title/Abstract]
#6	SMI [Title/Abstract]
#7	#4 OR #5 OR #6
#8	Randomized controlled trial [Title/Abstract]
#9	Clinical study [Title/Abstract]
#10	Controlled study [Title/Abstract]
#11	#8 OR #9 OR #10
#12	#3 AND #7 AND #11

In addition, for ongoing clinical trials, the following websites are analyzed, namely, the Chinese clinical registry (http://www.chictr.org/en/), National Institute of Health (NIH) clinical registry ClinicalTrials.gov (https://www.clinicaltrials.gov/) and the International Clinical Trials Registry Platform (http://www.who.int/ictrp/en/). Considering that there could be missing literature, representative journals are also manually searched, such as *China Journal of Traditional Chinese Medicine* and *Journal of Traditional Chinese Medicine.*

### 2.6 Study selection and data extraction

**Figure 1:**
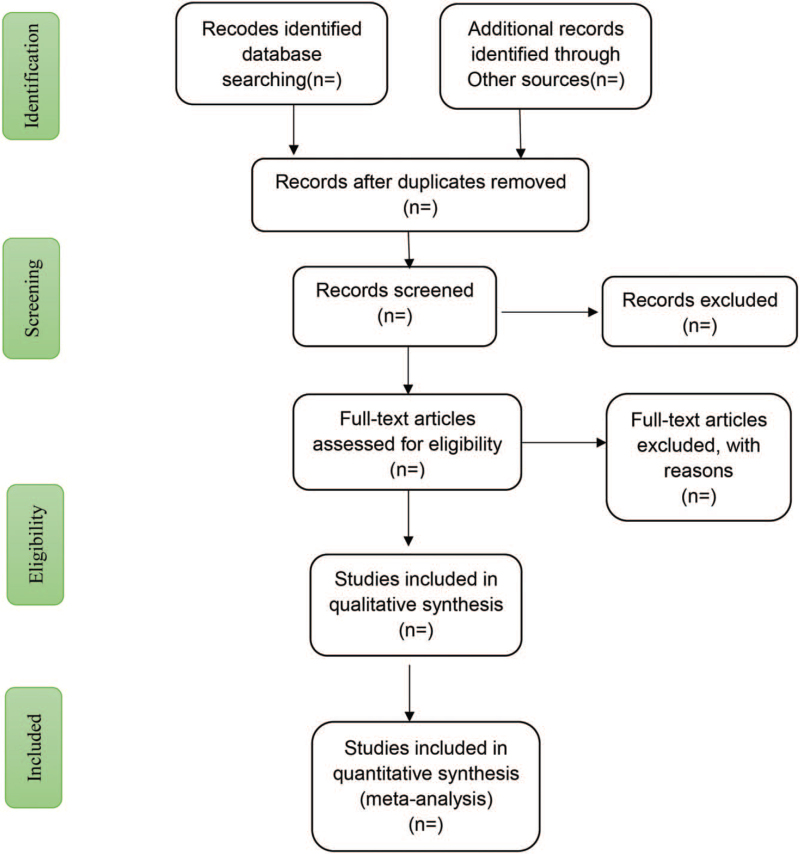
Flow diagram of study selection process.

Screening of literature by 2 independent researchers is carried out in accordance with pre-decided good inclusion criteria, initially focusing on literature titles topics for selecting literature to read, as preliminary screening. Consequently, it is necessary to read the full text for secondary screening. Whenever a consensus is not reached during the screening process, opinions are solicited from a third researcher, and an executive decision is finally obtained from them. The process of the selection is shown in Figure 1. Regarding data extraction, the research team prepares the extraction data table in advance, including:

*Investigation features:* first-author, study country, publication-year, title, journal, randomization method and blind method.
*Study patient features:* Inclusion/exclusion criteria, standard age, condition timespan, and sample size. *Intervention and control:* Type, dose, frequency and duration of intervention and control.
*Results:* endpoints, medical monitoring time-span, and side-effect incidences. Later, 2 researchers extract data independently, and cross-check will be required post-extraction, with any inconsistency solved by a third party. In addition, in the event of missing data, email is used communicate with article author to obtain data. If there is no reply, evaluation is performed depending upon the currently accessible datasets.

### 2.7 Assessment of risk of bias

Bias-risks pertaining to each investigation are separately evaluated through 2 researchers. Quality of individual studies is assessed through Cochrane Risk of Bias Assessment tool of Cochrane Reviewer's Handbook^[20]^ that includes 7 categories: random sequence generation, distribution, participant blindness, outcome evaluation blindness, incomplete outcome data, selective reporting, and other biases. Depending upon such categories, 3 judgments - including high-/low- and unclear-risk are given. Dataset outcomes for bias risk-assessment are represented by bias risk graphs.

### 2.8 Data synthesis and analysis

All data are statistically analyzed by Revman5.3. Hazard ratio is selected as the effect index for dichotomous variables and mean deviation was selected as the effect index for continuous variables. Datapoint projections and 95% confidence-intervals for every effect-size are determined. Heterogeneity is determined through standardized χ^2^ test (α = 0.1) and *I*^2^ test. Whenever *P* ≥ .1, and should *I*^2^ ≤ 50%, fixed-effects model is employed. Random-effects models are employed should *P* < .1 or *I*^2^ > 50%. Sub-cohort analyses are also conducted for probing reasons. Should heterogeneity be >75%, meta-analysis is not conducted. A narrative/qualitative summarization is consequently included.

### 2.9 Additional analyses

#### 2.9.1 Subgroup analysis

In order to probe probable reasons for heterogeneity, sub-cohort analyses are performed depending upon these pre-set sub-cohort assumptions.

- Baseline level (depending on data).
- Age (>70 years old or ≤70 years old).
- Treatment duration (≥2 weeks or <2 weeks).

#### 2.9.2 Sensitivity analysis

Sensitivity analysis is to re-estimate the combined effect size after removing a low-quality investigation and compare such results with those of the meta-analysis prior to exclusion, for probing investigation impact over combinatory effect-size dataset outcome robustness. Should there be no major variations in dataset outcomes following removal, this suggests low-sensitivity and reliable endpoints. Conversely, if large differences or even completely opposite conclusions are obtained post-exclusion, it indicates high sensitivity and low robustness for results. Therefore, it is necessary to be very cautious when interpreting such dataset outcomes, together with making conclusions, indicating the value and possible bias parameters linked to interventional route influences, together with further clarifying sources for controversy.

#### 2.9.3 Publication bias analysis

When the number of RCTs included in the study is ≥10, the report bias is assessed through constructing funnel-plot / Egger testing.

### 2.10 Grading the confidence of evidence

Grades of Recommendations Assessment Development and Evaluation profiler V.3.6 is employed for evaluating proof-quality, in line with Grades of Recommendations Assessment Development and Evaluation recommendations.^[21]^ Detailed assessment methodology consisted of the following:

Quality is enhanced, depending upon 3 parameters (residual confounding, dose–response gradient and large magnitude of effect), while quality is diminished through 5 parameters (study limitation/s, inconsistencies, no direction, publication-bias and imprecision). Qualities for accepted investigations are stratified as very low, low, moderate and high confidence level, accordingly.

## 3 Discussion

Sepsis is a serious inflammatory response syndrome, when local-to-systemic infection (and inflammatory immune responses) occur within this disorder, this consists as a main mortality-driver in patients with severe disease.^[2]^ Presently, there are many methods to avoid organ dysfunction caused by sepsis, using sepsis regulatory techniques such as liquid recovery, antibacterial treatment, vascular active medium, and support therapy. However, the prognosis of sepsis patients is still not ideal, with up to 40% in-hospital mortality rates.^[4]^ Consequently, many doctors and patients are actively exploring other complementary and alternative therapies for treatment. Although many studies have shown that SMI is effective in patients with sepsis. However, no high-quality literature has conducted a meta-analysis of the efficacy and safety of SMI for sepsis. Therefore, this study will systematically and objectively evaluate the effectiveness and safety of SMI in the treatment of sepsis patients. The conclusion of this review may bring benefit to patients with sepsis, clinicians and other relevant personnel. If the protocol is revised, the reasons of amendments will also be finally reported.

## 4 Ethics and dissemination

The protocol does not require ethical approval because it is intended only for ethical reviews that do not involve patient privacy data or animal experiments. The agreement is disseminated through peer-reviewed journals or conference reports.

## Acknowledgments

The authors would like to thank all the reviewers who participated in the review and MJEditor (www.mjeditor.com) for its linguistic assistance during the preparation of this manuscript.

PRISMA-P (Preferred Reporting Items for Systematic review and Meta-Analysis Protocols) checklist of this protocol is presented in online supplementary.

## Author contributions

**Formal analysis:** Ling Wang.

**Funding acquisition:** Linsheng Zeng.

**Methodology:** Yue Han, Guofu Zhong, Xiao Chang, Mingtai Chen, Linsheng Zeng, ling Wang.

**Project administration:** Guofu Zhong, Linsheng Zeng.

**Software:** Mujuan Xu.

**Supervision:** Mujuan Xu.

**Validation:** Xiao Chang, Mingtai Chen.

**Visualization:** Xiao Chang, Mingtai Chen.

**Writing – original draft:** Yue Han, Guofu Zhong, ling Wang.

**Writing – review & editing:** Yue Han, Guofu Zhong, ling Wang.
